# A Natural Language Processing Tool for Large-Scale Data Extraction from Echocardiography Reports

**DOI:** 10.1371/journal.pone.0153749

**Published:** 2016-04-28

**Authors:** Chinmoy Nath, Mazen S. Albaghdadi, Siddhartha R. Jonnalagadda

**Affiliations:** 1 Division of Health and Biomedical Informatics, Department of Preventive Medicine, Northwestern University Feinberg School of Medicine, Chicago, Illinois, United States of America; 2 Department of Medicine, Division of Cardiology, Northwestern University Feinberg School of Medicine, Chicago, Illinois, United States of America; Universidad de Castilla-La Mancha, SPAIN

## Abstract

Large volumes of data are continuously generated from clinical notes and diagnostic studies catalogued in electronic health records (EHRs). Echocardiography is one of the most commonly ordered diagnostic tests in cardiology. This study sought to explore the feasibility and reliability of using natural language processing (NLP) for large-scale and targeted extraction of multiple data elements from echocardiography reports. An NLP tool, EchoInfer, was developed to automatically extract data pertaining to cardiovascular structure and function from heterogeneously formatted echocardiographic data sources. EchoInfer was applied to echocardiography reports (2004 to 2013) available from 3 different on-going clinical research projects. EchoInfer analyzed 15,116 echocardiography reports from 1684 patients, and extracted 59 quantitative and 21 qualitative data elements per report. EchoInfer achieved a precision of 94.06%, a recall of 92.21%, and an F1-score of 93.12% across all 80 data elements in 50 reports. Physician review of 400 reports demonstrated that EchoInfer achieved a recall of 92–99.9% and a precision of >97% in four data elements, including three quantitative and one qualitative data element. Failure of EchoInfer to correctly identify or reject reported parameters was primarily related to non-standardized reporting of echocardiography data. EchoInfer provides a powerful and reliable NLP-based approach for the large-scale, targeted extraction of information from heterogeneous data sources. The use of EchoInfer may have implications for the clinical management and research analysis of patients undergoing echocardiographic evaluation.

## Introduction

Echocardiography is a widely used imaging modality for the diagnosis, management, and follow-up of cardiovascular disease (CVD) patients, and currently accounts for half of all cardiac imaging studies performed among Medicare beneficiaries [1]. Echocardiography provides key insights into the mechanisms of cardiovascular disease and therapeutic benefit of various interventions, while also providing a powerful research platform for the assessment of clinical trial enrollment eligibility and surrogate endpoints [2]. The interpretation of echocardiographic studies produces a continuous stream of large volumes of biomedical data found in non-standardized and/or heterogeneously organized clinical narrative reports. Public and private biomedical institutions have recognized the potential of using existing unstructured data sources, such as clinical trials, insurance programs, and electronic health records (EHRs) to improve patient phenotyping, guide clinical management and quality improvement initiatives, and support clinical research investigations [3]. However, because much of the data contained within echocardiography reports is often unstructured, it is not readily available for analysis [4].

A major barrier to leveraging unstructured data to improve patient care is the availability of tools that permit accurate extraction of high-quality data from the abundant and various types of unstructured data sources [5]. The financial cost and time-consuming nature of answering clinical questions through the manual extraction of structured data from clinical notes and other narratives is prohibitive [5,6]. Natural language processing (NLP) utilizes various algorithms to automatically extract and structure relevant clinical information from free text and semi-structured data sources [7–9]. The application of computational NLP techniques for extracting information from unstructured data in biomedical sources has the potential to impact both clinical practice and research [10]. Extensive use of computers and the Internet caused an exponential increase in patient information in clinical notes. Clinical notes contain peculiar drug names, anatomical nomenclature, other specialized names and phrases that are not standard in everyday English such as urinary incontinence, benign positional vertigo, l shoulder inj, po pain medications, a c5-6 acdf, st changes, resp status and o2 sats. There is also a high incidence of abbreviation usage and many of the abbreviations have a different meaning in other genres of English. For example: ASA (Acetyl Salicylic Acid, not as soon as), NAD (Nicotinamide Adenine Dinucleotide, here not no acute distress) and NC (No Change, not not clear). Generic information extraction resources and tools such as UMLS Metathesaurus [11], MedTagger [12], and cTAKES [13], which implement components such as synonym detector, tokenizer, sentence boundary detector, Part of Speech tagger [14], morphological analyzer [15], shallow parser [16], deep parser, named entity recognizer [17], association extractor, co-reference resolver [18], negation detector, temporality detector [19] and spelling corrector [15,20]. However, the shotgun NLP approach (10) of these systems misses a lot of concepts (low sensitivity) and produces many erroneous results (low precision or positive predictive value).

Prior studies of NLP-based echocardiographic data extraction from unstructured sources have been limited in scope, automation, and accuracy of data retrieval. We present an NLP-based technique capable of large-scale transformation of heterogeneous echocardiographic reports into a structured data format. We identified 80 data elements that are commonly evaluated in clinical practice and research studies using echocardiography, and applied our novel NLP-based extraction and processing algorithm, known as EchoInfer, to echocardiogram reports for automated extraction and organization. We show the feasibility and reliability of EchoInfer to transform three categories of data contained within an echocardiogram: (i) unstructured data, (ii) semi-structured data, and (iii) structured data, into a format that can be readily analyzed using conventional analytical approaches.

## Material and Methods

### Dataset and Study Population

EchoInfer was applied to *15*,*116* reports from *1683 adult* patients (1138 men and 545 women, mean age 67.9±13.88 years) undergoing echocardiography at a single academic medical center from 2004 and 2013. Echocardiography reports were obtained from clinical research studies approved by the Northwestern University Institutional Review Board, including a retrospective study conducted by one of the authors (MSA) to characterize echocardiographic metrics of valvular function in patients who received a bioprosthetic heart valve (from which the majority of reports were obtained). All “raw” reports used in this study were obtained from the Northwestern Medicine Enterprise Data Warehouse (NMEDW). The NMEDW is a single, integrated database of clinical and research data from all patients receiving treatment through Northwestern Medicine and its healthcare affiliates. The purpose of EDW is to collect, integrate and disseminate data to facilitate clinical research, quality, healthcare operations, and education [21].

### EchoInfer Extraction Approach

The reports processed by EchoInfer contained structured, semi-structured, and unstructured data across four different sections: (i) report text, (ii) procedure components, (iii) measurement and calculations, and (iv) conclusion or summary. The information for data elements was extracted from each section of the report and the output was then presented in a structured format for analysis.

Selection of echocardiographic data elements targeted for EchoInfer extraction was guided by the American Society of Echocardiography (ASE) recommendations for standardized echocardiography reporting [22], and the American College of Cardiology/American Heart Association (ACC/AHA) guideline statements on the use of echocardiography for the evaluation of valvular disease [23]. We identified eighty data elements of potential value to cardiovascular researchers contained within echocardiography reports and organized them into seven categories: (i) Left Ventricle, (ii) Right Ventricle, (iii) Aortic Valve, (iv) Mitral Valve, (v) Tricuspid and Pulmonic Valves, (vi) Atria, and (vii) Miscellaneous. These data elements are presented in Table 1.

**Table 1:** List of EchoInfer data elements targeted for extraction.

Left Ventricle	Right Ventricle	Aortic Valve	Mitral Valve	Tricuspid and Pulmonic Valves	Atria	Miscellaneous
Size: LVEDd and LVEDs	Size: basal dimension	Leaflet morphology: normal, thickened, or calcified	Leaflet morphology: normal, thickened, or calcified	Stenosis or Regurgitation severity: trace, mild, mild-moderate, moderate, moderate-severe, severe	Degree of LA or RA enlargement: mild, moderate, severe	Pericardial effusion size: no, trivial, small, moderate, large
Systolic function: LVEF	Function: TAPSE (cm) and RVEF (%)	Stenosis or Regurgitation severity: trace, mild, mild-moderate, moderate, moderate-severe, severe	Stenosis or Regurgitation severity: trace, mild, mild-moderate, moderate, moderate-severe, severe	Peak velocity (m/s)	LA diameter (cm)	IVC diameter (cm)
Diastolic function: GradeI, II, or III	Hypertrophy: present or absent	Peak velocity (m/s)	Peak velocity (m/s)	Mean velocity (m/s)	LA volume index (cm/m^2^)	Body surface area (m^2^)
Thickness type: concentric and/or basal septal	RVOT diameter (cm)	Mean velocity (m/s)	Mean velocity (m/s)	Peak gradient (mm Hg)	Right atrial pressure: 0–5, 5–10, or 15 mm Hg	
Septal thickness (cm)	RVOT VTI (cm)	Peak gradient (mm Hg)	Peak gradient (mm Hg)	Mean gradient (mm Hg)		
Degree of hypertrophy: mild, moderate, or severe	RVOT Peak Velocity (cm/s)	Mean gradient (mm Hg)	Mean gradient (mm Hg)	PASP (mm Hg)		
LV filling pressures: normal or increased	RVOT Mean velocity (cm/s)	LVOT diameter (cm)	E/A ratio			
E/e’ ratio	RVOT Peak gradient (mm Hg)	LVOT Peak velocity (cm/s)	MV VTI (cm)			
	RVOT Mean gradient (mm Hg)	LVOT Mean velocity (cm/s)	Pressure half-time (ms)			
		LVOT Peak gradient (mm Hg)	Effective regurgitant orifice area (mm^2^)			
		LVOT Mean gradient (mm Hg)	Regurgitant fraction			
		LVOT VTI (cm)	Mitral valve area (cm^2^)			
		AV VTI (cm)				
		Ao diameter (cm)				
		Aortic valve area (cm^2^)				
		Dimensionless index				
		Pressure half-time (ms)				
		Effective regurgitant orifice area (mm^2^)				
		Regurgitant fraction				
		Holodiastolic descending aortic flow reversal: present or absent				

Ao, aortic; AV, aortic valve; IVC, inferior vena cava; LA, left atrium; LVEDd and LVEDs, left ventricular end-dimension in diastole and systole, respectively; LVEF, left ventricular ejection fraction; LVOT, left ventricular outflow tract; MV, mitral valve; MR, mitral regurgitation, PASP, pulmonary artery systolic pressure; RA, right atrium; RVEF, right ventricular ejection fraction; RVOT, right ventricular outflow tract; TAPSE: tricuspid annular planar systolic excursion; TR, tricuspid regurgitation; VTI, velocity time integral.

Echocardiography reports contain many terms that can be used synonymously, such as ‘bioprosthetic heart valve’, ‘aortic prosthesis’, and ‘tissue valve prosthesis in the aortic position’, and a large number of abbreviations (e.g. AVA, MVA, E/e’, etc.). We selected representative example sentences and abbreviations to provide cues for the identification and extraction of the value of interest by the NLP algorithm implemented in EchoInfer. In addition to the example sentences, guidelines that constrain the range and type of values acceptable for the data elements were also provided (e.g. “Chamber thickness type values: concentric vs. basal septal”, “degree of hypertrophy values: mild, moderate, severe”, and “degree of septal thickness values: in centimeters”). These set of instructions provided a framework to automatically extract all relevant information accurately into a structured format.

### Designing EchoInfer

**Fig 1:**
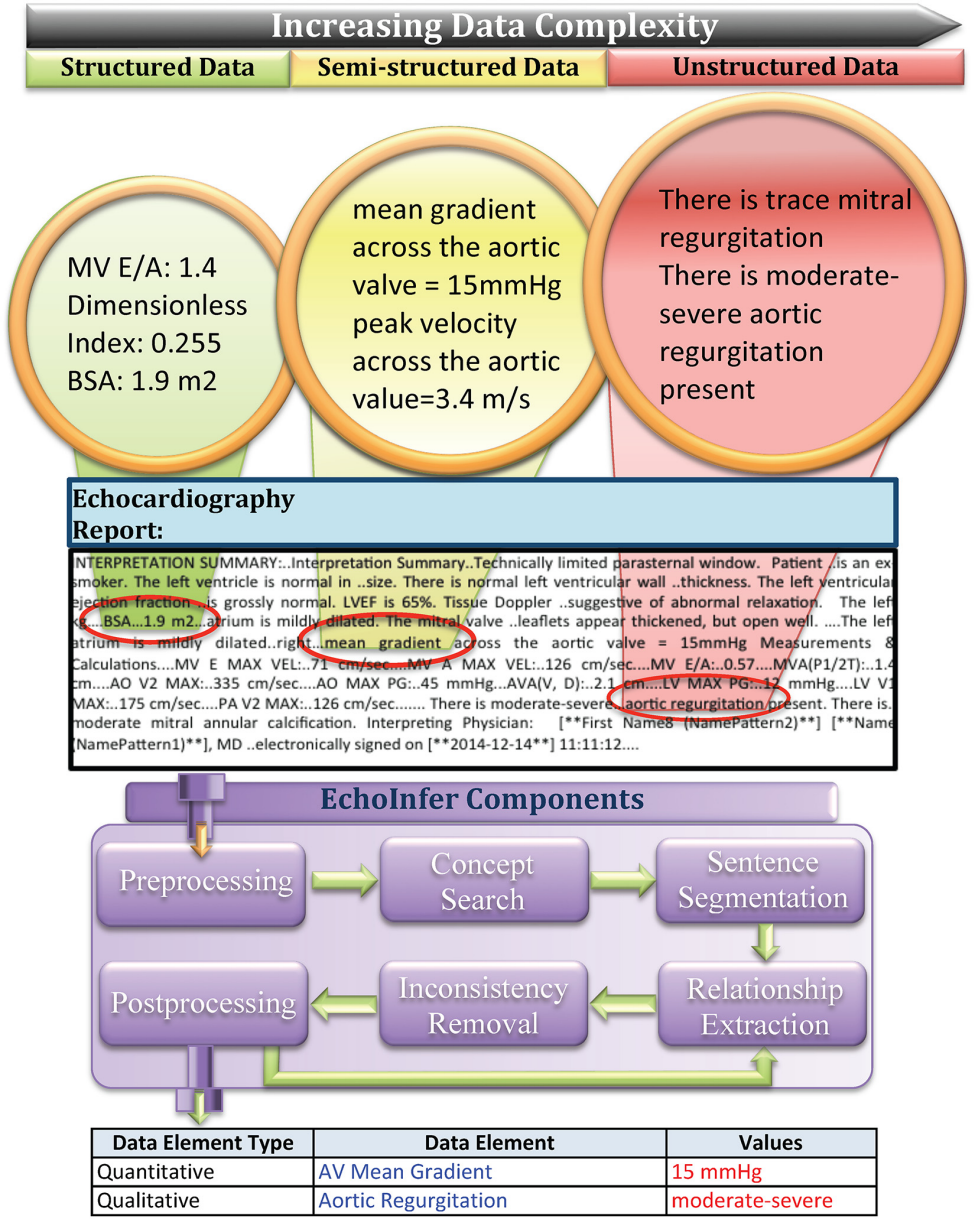
Extraction of data elements and values into structured format from structured, semi-structured, and unstructured data from echocardiography reports.

The NLP components used in constructing EchoInfer are as follows (Fig 1):

1. Pre-processing: Before processing, all reports were passed through a pre-processing step, to remove extra white spaces and ellipsis.

2. Document Selection: For each data element, reports mentioning the data element along with its value are identified. Data elements can be present in a report: (i) just once, or (ii) multiple times in different sections with the same value, or (iii) multiple times in different sections with multiple values. A rule-based engine was developed to identify all the mentions of each data element in the echocardiogram report.

3. Sentence Segmentation: Sentences were separated from each other in reports and the sentences that do not contain any data element were discarded. Special rules were built to distinguish between the following kinds of sentences: (i) structured (“MV E/A: ..0.57….MVA(P1/2T):..1.4 cm….AO V2 MAX:..335 cm/sec….”), (ii) semi-structured (“Peak.. velocity across the aortic valve = 5.0 m/s”) and (iii) unstructured (“The forward flow across the aortic valve is ..increased at 3.4 m/s with a mean gradient of 28 mm.. Hg”). The extra ‘.’ symbols in each of the above examples, which are caused by errors in the way the notes are stored in the EHR database, are ignored automatically to correctly capture the complete sentence in each case.

In certain cases, an unstructured sentence contained a data element but a related value was mentioned after one or two sentences (for example, Sentence 1: “A bioprosthetic valve is seen in the aortic position, which is well seated.” Sentence 2: “The…peak velocity across this valve is 1.9 m/second.” Sentence 3: “The mean gradient is 16 mmHg ..and peak gradient is 27 mmHg.”), a scenario which has previously been described as co-reference relation. Jonnalagadda et al [24] previously constructed a system that determines similar co-reference relations (whether two mentions refer to the same entity or not), which we implemented here to improve the sensitivity of our EchoInfer algorithm.

4. Relationship Extraction: A set of regular expressions that consist of concise rules around patterns of strings and characters were developed to extract cardiac parameters in the form of data elements [25–27]. We first extracted both the text spans containing both the data element and values of interest. For example, using the regular expression, (“.{30} (\\sava\\s|aortic\\s*valve\\s*area|\\sav\\sarea).{100}”), we identified all thirty characters before and hundred characters after the data element “AVA” (that can be referred to as “AVA”, “aortic valve area”, or “AV area”) for relationship extraction. An additional set of regular expressions was used to extract categorical and numerical values for the respective data elements within or corresponding to the targeted text span. In many cases it was found that the rules are generalizable across data elements (for example, AV peak velocity and MV peak velocity). Hence, we also developed generalizable regular expressions that accept the various synonyms of the regular expression as parameters. For further details, we provided list of words, phrases and synonyms used by EchoInfer for each data elements (see S1 Table).

5. Filtering of quantitative echocardiographic data:

(a) Estimation of burden of missing data: Some values of data elements in the reports were unspecified (E.g. “data elements without values: Pressure half time…??, Leaflet Morphology… [**Name (NamePattern1)**]). EchoInfer quantified the number of reports without any mention of the targeted data value to provide an estimate of the burden of missing data and potential bias in a given data set.

(b) Data inconsistency removal: In more than 90% of the cases, the values of each data element were consistent with the range and type of values that can be accepted for the data element. For example, the aortic valve area or “AVA” can expressed in cm2 or sq. cm but not in mmHg or cm/sec. However, different inconsistencies existed in the echocardiographic reports and filtering method was applied to resolve this. (i) EchoInfer identified typing errors such as incorrect units of measurement (e.g. “MV mean gradient …21.54 cm/s”); no unit of measure (e.g. “AV peak velocity …2.54”) and flagged them for removal or estimation from other values, (ii) EchoInfer also identified systematic errors, where values were reported with dissimilar but consistent units (e.g. cm/s, m/sec and mm/s), (iii) homogenization of dimensional units were implemented wherever applicable (e.g. convert mm to cm, cm2 to m2 and cm/second to m/second), (iv) anatomically and physiologically inconsistent values were identified and flagged for removal using the following constraints: systolic dimensions are smaller than diastolic dimensions (e.g. LVEDs < LVEDd), all peak velocities are less than peak gradients (e.g. MV peak velocity < MV peak gradient), and all mean gradients are less than peak gradients (e.g. MV mean gradient < MV peak gradient), and (v) outliers were identified based on the interquartile range for each qualitative data elements and outliers outside the range (e.g. “septal thickness…1.2 m”), were removed [28].

6. Post-processing: After the output is automatically populated into a structured format as seen in Fig 1, the post-processing component performed additional operations to ensure removal of continuous values and calculating means in the event where ranges of values may exist (e.g. 25 to 30 mmHg).

### EchoInfer Evaluation Methods

Training set: A set of 100 reports were randomly selected and further divided into five batches comprising of 20 reports. We used an initial set of regular expressions and rules for each data element to train the system for the first batch. After reviewing successive batches from the training subset, the regular expressions and rules were improved to maximize the accuracy. We identified instances where existing rules failed to extract information and therefore made further modification to the pattern sets. These steps were repeated, updating the pattern iteratively until the system extracted all possible instances of data elements. We also incorporated additional synonyms and abbreviations to the pre-defined list of data elements as needed. In the next step, a cardiologist (MSA) evaluated EchoInfer’s performance and further iterations were developed to improve the sensitivity and specificity of extraction. The performance of the initial pattern set was found suboptimal, wherein sensitivity (recall) and positive predictive value (precision) were less than 70%. Five to six iterations of the regular expressions pattern set development were performed until we the sensitivity (recall), positive predictive value (precision) and F1-score (the harmonic mean of sensitivity and positive predictive value) at least 90% [29].

Test set and Accuracy Assessment: Once the patterns and rules were developed using the training set, it was further validated using another randomly obtained test set of fifty reports. In order to obtain the accuracy of our system, two stages of manual reviews were performed. In the first step, fifty randomly selected echocardiogram reports from the overall cohort were annotated to validate for eighty data elements, i.e. 50×80 = 4000 data element values. Two annotators (CN and SRJ) evaluated each report manually and consulted a third annotator (MSA) to resolve any inconsistencies present. In the second step, two annotators selected 200 random reports for patients different from the previous test and analyzed only two data elements (mean aortic valve gradient and aortic regurgitation). Similarly, from another set of 200 randomly selected reports related to different patients, two more data elements were analyzed (left ventricular ejection fraction (LVEF) and left atrial diameter). In evaluating the performance of the NLP algorithm in EchoInfer, the annotators determined the sensitivity, specificity, positive predictive value, negative predictive value, and F1-score for all extracted data elements.

The performance of EchoInfer was also validated on multiple sets of independent reports. In one of the validation process, 100 reports were randomly drawn from a separate clinical research study cohort at our institution (known as ARDS) and the following 10 data elements were randomly selected for evaluation of EchoInfer’s performance: LVEF, degree of left ventricle hypertrophy, grade of diastolic dysfunction, left atrial diameter, left ventricular outflow tract (LVOT) peak velocity, LVOT diameter, mitral valve mean gradient, aortic root diameter, tricuspid regurgitation peak gradient, and body surface area. In another validation step, 100 echocardiogram reports were randomly selected from another research cohort (PARAGON-HF) to validate ten different data elements: AVA, AV peak gradient, tricuspid regurgitation peak gradient, LVOT peak gradient, AV velocity time integral, LVOT velocity time integral, degree of left ventricular hypertrophy, qualitative grade of aortic stenosis, mitral stenosis, and mitral regurgitation.

The final output consisting of the targeted data elements for extraction with their respective values was generated in a predefined structured format to facilitate statistical analyses. For each report and for all eighty data elements, the output included the sentence segment containing the data element with values, the list of all values present in report for that data element, and summary statistics such as the number of values in the report for that data element, maximum value, minimum value and the last value mentioned in the echocardiogram report. Examples of such sentence segments for different data elements are shown in Table 2. The output of the EchoInfer NLP algorithms were presented in a structured format to the investigators of a retrospective study of surveillance of BHV function post-operatively over the span of 10 years using the echocardiogram reports described above.

**Table 2:** Examples of EchoInfer’s identification of data element and corresponding value structured output.

Data Elements	Text Span for Information Extraction	Output
AV mean Gradient	…the mean gradient across the aortic valve is 25–30 mmhg.	27.5 mmhg
Aortic regurgitation	…moderate-severe aortic regurgitation is present.	moderate-severe

The datasets used for the study were approved for information extraction research by Northwestern University Institutional Review Board (STU00201723, STU00201246, and STU00068198). We obtained waivers for HIPAA authorization, consent process, and consent documentation since the research only involves rendering structure to existing data, documents, records, or specimens. All the data is archived in encrypted school of medicine servers after analysis.

## Results

### Echocardiographic Report Characteristics

The 15,116 reports included 3,456 transesophageal echocardiographic reports, 1,050 transthoracic echocardiographic reports, 861 stress echocardiographic reports, and 10,590 doppler echocardiographic reports for this study. EchoInfer evaluated 3,725 reports from patients with a history of aortic valve replacement (AVR), 828 reports from patients with a history of mitral valve (MV) replacement, 441 reports from patients with a history of mitral valve repair, 677 reports from patients with combined AVR and MV replacement or repair, and 9,444 reports from patients for various indications without a history of valvular surgery.

### Performance of EchoInfer

The total time taken by EchoInfer system for analyzing 15,116 echocardiogram reports and generating output in a structured format was less than an hour on a personal laptop. For the initial evaluation, fifty reports randomly selected as the ‘test set’ were compared with the output of EchoInfer to validate eighty data elements. EchoInfer achieved an overall precision (positive predictive value) of 94.06%, overall recall (sensitivity) of 92.21%, and overall F1-score 93.12%. We found a similarly high level of accuracy for EchoInfer’s output for all data elements (S2 Table). Table 3 shows the ten most commonly occurring data elements in the echocardiography reports and associated performance of EchoInfer with F1-Scores ranging from 91.80% to 96.10%.

**Table 3:** Precision and Recall for ten most frequent data elements identified in 15,116 echocardiograms.

	Data Elements	Precision %	Recall%	F1-Score %
	Overall	94.06	92.21	93.12
1	TRICUSPID REGURGITATION	92.3	94.73	93.51
2	LVEF	95.65	93.62	94.62
3	AO ROOT DIAMETER	97.67	95.45	96.55
4	AV MEAN GRADIENT	95.12	92.86	93.98
5	MITRAL REGURGITATION (no trace, trivial, mild, moderate, severe)	93.02	95.24	94.12
6	MITRAL LEAFLET	97.37	94.87	96.10
7	BODY SURFACE AREA	97.37	97.37	97.37
8	AORTIC REGURGITATION	94.12	91.43	92.75
9	AV PEAK GRADIENT	93.75	96.77	95.24
10	AV PEAK VELOCITY	93.33	90.32	91.80

In a second manual review, one of the authors (MA, a cardiologist) manually verified both quantitative (mean aortic gradient, LVEF, left atrial dimension) and qualitative (aortic regurgitation grade) values obtained from another randomly selected 400 echocardiogram reports from 373 different patients. The precision (positive predictive value) and recall (sensitivity) of EchoInfer for the extraction of mean aortic valve gradient was 98.05% and 94.40%, LVEF 97.40% and 94.90%, aortic regurgitation 99.90% and 99.65%, and left atrial dimension 98.90% and 98.10%, respectively (Table 4).

**Table 4:** Summary on precision and recall for 21 different random data elements validated on multiple data sets of echocardiographic reports.

Set Name*	Data Elements	Recall	Precision	Note
ARDS	**LVEF**, **DEGREE OF LV HYPERTROPHY**, DIASTOLIC DYSFUNCTION, **LA DIMENSION**, LVOT PEAK VELOCITY, MV MEAN GRADIENT, BSA, LVOT DIAMETER, TR PEAK VELOCITY, AO ROOT DIAMETER	95–99.9%	> 96%	10 data elements, tested on 100 random reports selected from ARDS project.
PARAGON-HF	AVA, AV PEAK GRADIENT, TR PEAK GRADIENT, LVOT PEAK GRADIENT, AV VTI, LVOT VTI, AORTIC STENOSIS, MITRAL STENOSIS, MITRAL REGURGITATION, **DEGREE OF LV HYPERTROPHY**	92–99.9%	> 98%	10 data elements, tested on 100 random reports selected from Paragon project.
EDW_SET#1	AV MEAN GRADIENT, AORTIC REGURGITATION	92–99.9%	> 97%	2 data elements, tested on 200 random reports from present study
EDW_SET#2	**LVEF**, **LA DIMENSION**	94–99.9%	> 98%	2 data elements, tested on 200 random reports from present study

*Data elements in bold signifies, data element tested on multiple data sets.

**Fig 2:**
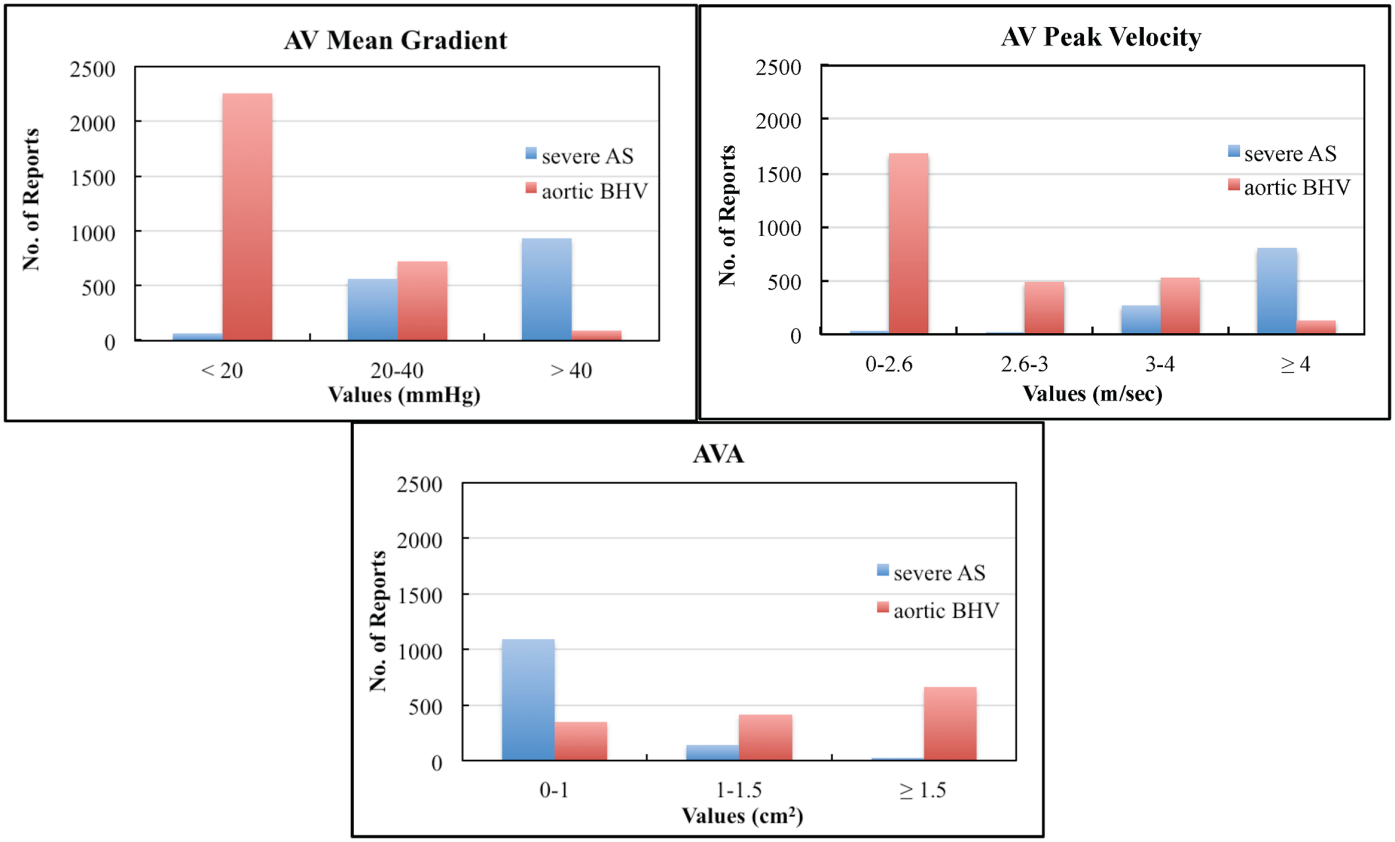
Number of reports containing specified ranges of values from patients identified by EchoInfer as having either severe aortic stenosis (AS) or an aortic bioprosthetic heart valve (BHV) demonstrating the expected pattern for aortic valve (AV) mean gradient, AV peak velocity, and aortic valve area (AVA).

Since echocardiogram reports were de-identified, we were unable to ascertain the exact date of surgery or identity of pre- or post-operative echocardiograms. However, a frequency histogram of mean AV mean gradients, AV peak velocity and AVA from patients with a history of severe aortic stenosis and a history of AVR demonstrates the expected reduction in aortic gradients associated with a clinical history of valve replacement (Fig 2).

In an effort to explore the generalizability of the NLP algorithm developed for EchoInfer in patients with a range of medical conditions and indications for echocardiography, we applied EchoInfer to clinical research cohorts without known valvular heart disease. The accuracy of EchoInfer for the extraction of 19 unique data elements was assessed in 100 reports from a study of patients with heart failure with preserved ejection fraction (PARAGON-HF) and in 100 reports from study of patients with acute respiratory distress syndrome (ARDS). The sensitivity (recall) and positive predictive value was above 92% and 96%, respectively in the combined cohorts (Table 4).

During the validation of EchoInfer, we found that some rules failed to include alternative abbreviations and synonyms. For example, the abbreviations “AO Max PG” for aortic valve peak gradient, and “AR or AI” for aortic regurgitation/insufficiency were not included in the initial set of rules. EchoInfer was tasked with handling a variety of linguistic variants in order to recognize, extract, and categorize the desired data elements, and the necessary changes were made in the NLP approach to accommodate the variable expression of various data elements of interest (Table 5, also see S1 Table).

**Table 5:** Examples showing various synonymous terminologies used in echocardiography reports for data elements targeted for EchoInfer extraction.

aortic valve peak gradient	mitral valve peak velocity
Ao max pg	MV peak velocity
AV peak gradient	mitral valve peak velocity
aortic valve peak gradient	MV peak recorded velocity
AV peak pressure gradient	mitral peak recorded velocity
aortic valve peak pressure gradient	peak velocity across MV
peak pressure gradient across aortic valve	peak velocity across mitral valve
peak pressure gradient across aortic bioprosthetic valve	peak velocity across mitral bioprosthetic valve
peak pressure gradient across aortic bioprosthesis	peak velocity across bioprosthetic mitral valve
Ao peak pressure forward flow gradient	peak velocity across mitral bioprosthesis
aortic valve peak pressure forward flow gradient	across mitral bioprosthetic valve peak velocity
peak transaortic valve gradient	across bioprosthetic mitral valve peak velocity
peak trans aortic valve pressure gradient	across mitral bioprosthesis peak velocity
peak Ao valve gradient	peak transmitral velocity
peak aortic valve gradient	peak mitral valve velocity
peak Ao pressure difference	peak mitral velocity

Although EchoInfer achieved a degree of sensitivity, examples where EchoInfer’s NLP algorithms failed to extract the targeted data element value are shown in Table 6. Most of these cases were (a) related to the disparate phrasing of the data elements, (b) uncommon characters around data elements or values, and (c) incidents of misspelling of terms. EchoInfer was designed to avoid overly sensitive NLP algorithm rules that would come at the expense of reduced specificity and precision in echocardiograph reports. Hence, we limited the number of non-generalizable rules to the existing set to avoid over-fitting.

**Table 6:** Examples of non-standardized echocardiographic reporting that are not identified or extracted by EchoInfer.

Examples: EchoInfer Failed to Extract	Reason
The ..velocity across the AV bioprosthesis has increased.. from 1.6 m/s--> 2.0 m/s	uncommon characters “-->”
A bioprosthetic valve ..is present in the aortic position. Maximum ..gradient of 24 mm Hg, mean 13 mm Hg	mean gradient phrase is missing
The .. forward flow across the bopprosthetic valve is 3.7 m/s with a mean .. gradient of 30 mm Hg	misspelling of bioprosthetic
across the aortic valve is increased with a mean gradient of [**12–02**] mmhg	uncommon characters around digits
ava 0.53 am2. (ava index is 0.3 cm2/m2) dimensionless index (tvi ratio) = 0.19	misspelling of dimension cm2
there is a well seated, ..well functioning stentless porcine aortic valve. There.. is no significant stenosis or regurgitation of the ..prosthesis	rare phrasing of aortic stenosis

## Discussion

We have developed an automated NLP-based system (EchoInfer) for the targeted extraction and processing of heterogeneous data, and demonstrated the feasibility of using EchoInfer to extract a large-scale set of eighty data elements related to cardiac structure and function contained within echocardiographic reports. EchoInfer was able to identify clinically relevant echocardiographic measurements catalogued in narrative echocardiogram reports with a high degree of sensitivity and specificity compared to manual review. The automated extraction technique processed parameters related to valvular stenosis and regurgitation, ventricular size and function, atrial size, and hemodynamics with a high degree of accuracy. Valvular gradients, velocities, and peak areas extracted using EchoInfer were biologically consistent with a clinical history of severe aortic stenosis and AVR supporting the potential of our NLP-based tool as a clinical and research tool. Additionally, EchoInfer provides an assessment of the number of reports not containing data values of interest, which can provide investigators with a rapid and automated tool for evaluating the burden of missing data within a data set and the potential for bias in answering a variety of research questions (see S3 Table).

The ability to rapidly use clinical data contained within electronic health records, including clinical notes and imaging studies, has wide ranging implications for the risk stratification, management, and research of patients with CVD and other diseases. Automated methods for the extraction of data elements to improve patient phenotyping have been studied in various diseases and implemented in diverse data settings [13,30–34]. However, only a few studies have utilized NLP for the extraction and organization of data from echocardiographic reports [35–37]. Several limitations exist with respect to the extent and accuracy of echocardiographic parameters extracted using the NLP-based techniques reported in these studies. Garvin et al [35] used a NLP system to extract an isolated echocardiographic parameter of ventricular function: left ventricular ejection fraction (LVEF). Although their automated extraction technique functioned with acceptable accuracy (positive predictive value = 95% and F1-score = 92%), the extracted information was limited to a single parameter of ventricular function without providing important information regarding cardiac structure or other metrics of ventricular function. Similarly, other reports of echocardiographic NLP algorithms have been limited by the relatively small number of data elements extracted with poor sensitivity [36], and lack of standardized evaluation of NLP performance [38]. Wells et al [9] provided an assessment of cardiac structure, including LV size in end-diastole and end-systole, LV septal and posterior wall thickness, left atrial diameter, and aortic diameter. However, the extraction method implemented was not fully automated and was limited to the extraction of data from structured data fields (i.e., not free text). In comparison, the accuracy of EchoInfer exceeds that of reported studies, extracts significantly more comprehensive information regarding cardiac structure, function, and hemodynamics, and has the capability to extract echocardiographic information from structured, semi-structured, and unstructured data sources.

EchoInfer performed comparably well in three different clinical studies with diverse patient populations: a) a study examining myocardial recovery after initial hospitalization for heart failure in patients with non-ischemic cardiomyopathy—where it extracted LVEF values, b) a study of the efficacy and safety of LCZ696 in heart failure patients with preserved ejection fraction (PARAGON-HF)–where it extracted LVEF, BMI, and age values, and c) a novel study of an implementation and social network strategy for acute pulmonary illnesses—where it extracted LVEF, LA dimension, LA volume, LV hypertrophy, and diastolic dysfunction values. Future studies will include further evaluation in other clinical research settings, and exploring the possibility of using such algorithms for automated surveillance of the EHR to monitor changes in patients’ health status. Additionally, future work will investigate automatic correction of data inconsistencies that would obviate their removal and therefore produce a richer data set.

There are some limitations in this study. Although EchoInfer is highly accurate for the extraction of desired echocardiographic data elements, the extracted data may need to be manually reviewed to assess its utility in clinical or research applications. The organization of echocardiographic reports at our institution provides an instructive example where the sequence of structured, semi-structured, and unstructured sections occurred in a different order within the echocardiographic reports during the time period of retrospective data extraction. Prior to around 2008, the reports positioned the most unstructured text (i.e., the conclusion) at the top of the body of the report. Thereafter, the conclusion was placed at the end of the report. Since the most definitive statements regarding echocardiographic data elements of interest (for example, mean aortic valve gradient) are often located within the conclusion, we could not simply design EchoInfer to extract the first or last mention of the data element as the highest fidelity value. Thus, for clinical purposes, understanding of context may be necessary to identify the “true value” of a data element when the variability in the value of the data element is deemed to be clinically significant (>20% variability in all extracted mean aortic valve gradients from a single report, for example). Alternatively, the average or most commonly reported (i.e., mode) of all values for a particular data element could also be used depending upon the clinical scenario or research question.

Another limitation is that the extraction of data elements with qualitative values may be problematic. These definitions may vary between institutions and over time and are prone to data entry errors. However, EchoInfer extracts many of the necessary quantitative parameters that are used to inform the clinical contextualization of these qualitative statements and minimize the chance of a biologic inconsistent value being used for analysis that was generated by the errant entry of an inaccurate qualitative comment by the echocardiographer. For example, a patient is unlikely to have “severe left atrial (LA) enlargement” when all extracted LA dimensions (i.e., quantitative metrics of diameter and volume) are consistent with mild LA enlargement.

Next, EchoInfer was tested at a single institution with relatively stable content of echocardiography reports over the study period. However, as mentioned above, the organization of echocardiography reports at our institution evolved over the study period varied thereby supporting the ability of EchoInfer to manage heterogeneous data formats. Many institutions and clinical trial databases containing echocardiographic data may use structured databases that prospectively collect various (mostly quantitative) data from echocardiographic reports. In such cases, EchoInfer could be used as a supplementary tool to facilitate extraction of data that was not collected prospectively. Additionally, we have openly shared the EchoInfer system with other researchers, where the performance of this tool can be studied in diverse clinical and research settings. Although EchoInfer may be susceptible to transcription errors, misspelling, measurement unit inconsistencies, and non-standardized expressions of echocardiographic parameters, these instances were limited in number and did not have a large impact on the accuracy of EchoInfer in our experience. At Northwestern University, we are using EchoInfer in conjunction with generic information extraction resources and tools such as UMLS Metathesaurus [11], MedTagger [12], and cTAKES [13]. This allows us to combine EchoInfer’s highly accurate performance for echocardiography notes with the shotgun NLP approach [10] of other systems that extract large volume of concepts across other narratives in EHR.

Lastly, the heterogeneity of echocardiography reports analyzed by EchoInfer may have been limited by the inclusion/exclusion criteria of our study cohorts, including lack of reports from patients with mechanical valves. However, we have demonstrated that EchoInfer can function with a comparable degree of accuracy in patient cohorts from three different clinical research studies suggesting broad applicability of EchoInfer’s NLP algorithms.

## Conclusions

EchoInfer’s NLP algorithms permit large-scale extraction of structured data pertaining to cardiovascular structure and function from heterogeneously organized echocardiographic narratives and reports contained within the EHR. EchoInfer is capable of automated, rapid, and accurate extraction of a multitude of cardiac structure, function, and hemodynamic parameters from echocardiographic reports that may have potential applications in clinical practice and research studies.

## Supporting Information

S1 Table List of eighty data elements with synonymous terminologies. This table lists the eighty data elements that EchoInfer extracts from Echo reports along with their synonyms.
(DOC)

S2 Table Accuracy of eighty data elements extracted during this study. This table lists the accuracy (precision, recall, specificity, negative predictive value and F-1 score) of the eighty data elements extracted by EchoInfer.
(DOC)

S3 Table Breakdown of percentage of echocardiographic reports lacking any mention of various data elements as determined by EchoInfer. This figure shows the relative proportion of Echo reports that miss ten of eighty data elements.
(DOC)
