# Supplementary material for: A Natural Language Processing Tool for Large-Scale Data Extraction from Echocardiography Reports
Source: PLoS One. 2016 Apr 28;11(4):e0153749. doi: 10.1371/journal.pone.0153749 (PMC4849652; doi:10.1371/journal.pone.0153749)
Supplement: S1 Table — This table lists the eighty data elements that EchoInfer extracts from Echo reports along with their synonyms. (DOC) [file pone.0153749.s001.doc]

| **S.No** | **Data** **Elements** | **Synonymous** **Terminologies** | | | | | |
| --- | --- | --- | --- | --- | --- | --- | --- |
| **1** | AVA | AVA | Aortic Valve Area | AV area |  |  |  |
| **2** | MVA | MVA | Mitral Valve Area | MV area |  |  |  |
| **3** | AORTIC LEAFLET | Aortic Valve leaflets | Aortic Valve trileaflet | Aortic valve |  |  |  |
| **4** | MITRAL LEAFLET | Mitral Valve leaflets | Mitral Valve trileaflet | Mitral Valve |  |  |  |
| **5** | AV PEAK GRADIENT | peak pressure gradient across aortic bioprosthetic valve | peak pressure gradient across aortic bioprosthesis | Ao peak pressure forward flow gradient | aortic valve peak pressure forward flow gradient | peak transaortic valve gradient | peak trans aortic valve pressure gradient |
| **5** | AV PEAK GRADIENT | aortic valve peak gradient | AV peak pressure gradient | aortic valve peak pressure gradient | peak pressure gradient across aortic valve | peak Ao valve gradient | peak aortic valve gradient |
| **5** | AV PEAK GRADIENT | peak Ao pressure difference | Ao max pg | AV peak gradient | AV peak/mean gradient | peak/mean aortic gradient |  |
| **6** | MV PEAK GRADIENT | peak pressure gradient across mitral bioprosthetic valve | peak pressure gradient across mitral bioprosthesis | MV peak pressure forward flow gradient | mitral valve peak pressure forward flow gradient | peak transmitral valve gradient | peak trans mitral valve pressure gradient |
| **6** | MV PEAK GRADIENT | mitral valve peak gradient | MV peak pressure gradient | Mitral valve peak pressure gradient | peak pressure gradient across mitral valve | peak MV valve gradient | peak mitral valve gradient |
| **6** | MV PEAK GRADIENT | peak MV pressure difference | MV max pg | MV peak gradient | MV peak/mean gradient | peak/mean mitral gradient |  |
| **7** | MR PEAK GRADIENT | MR max pg | MR peak pressure gradient | MR peak gradient |  |  |  |
| **8** | TR PEAK GRADIENT | TV max pg | TR peak pressure gradient | TR peak gradient |  |  |  |
| **9** | TV PEAK GRADIENT | PV max pg | TV peak pressure gradient | TV peak gradient |  |  |  |
| **10** | PV PEAK GRADIENT | TR max pg | PV peak pressure gradient | PV peak gradient |  |  |  |
| **11** | LVOT PEAK GRADIENT | LVOT peak pressure gradient | LVOT peak gradient | LVOT with….peak pressure gradient | LVOT with…peak gradient |  |  |
| **S.No** | **Data Elements** | **Synonymous Terminologies** | | | | | |
| **13** | AV MEAN GRADIENT | across bioprosthetic valve in aortic position …mean gradient | Aortic valve mean pressure gradient | Aortic Valve mean gradient | AV mean pressure gradient | AV mean gradient | mean pressure gradient across aortic valve |
| **13** | AV MEAN GRADIENT | mean pressure difference across aortic valve | across aortic valve mean pressure gradient | mean gradient across aortic prosthesis | AO mean pg | mean transaortic valve pressure gradient | mean AV pressure gradient |
| **14** | MV MEAN GRADIENT | across bioprosthetic valve in mitral position …mean gradient | Mitral valve mean pressure gradient | Mitral Valve mean gradient | MV mean pressure gradient | MV mean gradient | mean pressure gradient across mitral valve |
| **14** | MV MEAN GRADIENT | mean pressure difference across mitral valve | across mitral valve mean pressure gradient | mean gradient across mitral prosthesis | mean transmitral valve pressure gradient | mean MV pressure gradient |  |
| **15** | PV MEAN GRADIENT | PV peak pressure gradient | PV peak gradient | Pulmonary valve with….peak pressure gradient | Pulmonary valve with…peak gradient |  |  |
| **16** | TRICUSPID MEAN GRADIENT | mean gradient across the Tricuspid valve | mean pressure gradient across the Tricuspid valve | across the Tricuspid valve mean pressure gradient | across the Tricuspid valve mean gradient | Tricuspid mean gradient | Tricuspid mean pressure gradient |
| **16** | TRICUSPID MEAN GRADIENT | TV mean gradient | TV mean pressure gradient |  |  |  |  |
| **17** | RVOT PEAK VELOCITY | peak recorded velocity across RVOT | across the RVOT peak velocity | across RVOT peak recorded peak velocity | RVOT peak recorded velocity | RVOT peak velocity | peak RVOT velocity |
| **17** | RVOT PEAK VELOCITY | RVOT peak velocity |  |  |  |  |  |
| **18** | LVOT PEAK VELOCITY | peak recorded velocity across LVOT | across the LVOT peak velocity | across LVOT peak recorded peak velocity | LVOT with….peak recorded velocity | LVOT peak velocity | peak LVOT velocity |

| **S.No** | **Data** **Elements** | **Synonymous Terminologies** | | | | | |
| --- | --- | --- | --- | --- | --- | --- | --- |
| **18** | LVOT PEAK VELOCITY | peak recorded velocity across LVOT | across the LVOT peak velocity | across LVOT peak recorded peak velocity | LVOT with….peak recorded velocity | LVOT peak velocity | peak LVOT velocity |
| **18** | LVOT PEAK VELOCITY | LVOT peak velocity | LVOT peak recorded velocity |  |  |  |  |
| **19** | TV PEAK VELOCITY | peak recorded velocity across the tricuspid valve | peak velocity across the tricuspid valve | across the tricuspid valve peak velocity | across the tricuspid valve peak recorded peak velocity | Tricuspid valve peak velocity | peak Tricuspid valve velocity |
| **19** | TV PEAK VELOCITY | TV peak velocity | TV peak recorded velocity |  |  |  |  |
| **20** | MV PEAK VELOCITY | peak velocity across mitral bioprosthetic valve | peak velocity across bioprosthetic mitral valve | peak velocity across mitral bioprosthesis | across mitral bioprosthetic valve peak velocity | across bioprosthetic mitral valve peak velocity | across mitral bioprosthesis peak velocity |
| **20** | MV PEAK VELOCITY | peak transmitral velocity | peak mitral valve velocity | peak mitral velocity | MV peak velocity | mitral valve peak velocity | MV peak recorded velocity |
| **20** | MV PEAK VELOCITY | mitral peak recorded velocity | peak velocity across MV | peak velocity across mitral valve |  |  |  |
| **21** | AV PEAK VELOCITY | peak velocity across aortic bioprosthetic valve | peak velocity across bioprosthetic aortic valve | peak velocity across aortic bioprosthesis | across aortic bioprosthetic valve peak velocity | across bioprosthetic aortic valve peak velocity | across aortic bioprosthesis peak velocity |
| **21** | AV PEAK VELOCITY | peak transaortic velocity | peak aortic valve velocity | peak aortic velocity | AV peak velocity | Aortic valve peak velocity | AV peak recorded velocity |
| **21** | AV PEAK VELOCITY | Aortic peak recorded velocity | peak velocity across AV | peak velocity across aortic valve |  |  |  |
| **22** | PV PEAK VELOCITY | peak recorded velocity across the pulmonary valve | peak velocity across the pulmonary valve | across the pulmonary valve peak velocity | across the pulmonary valve peak recorded peak velocity | Pulmonary valve peak velocity | peak Pulmonary valve velocity |
| **22** | PV PEAK VELOCITY | PV peak velocity | PV peak recorded velocity |  |  |  |  |

| **S.No** | **Data** **Elements** | **Synonymous** **Terminologies** | | | | | |
| --- | --- | --- | --- | --- | --- | --- | --- |
| **2**3 | TR PEAK VELOCITY | TR peak velocity | TR max velocity |  |  |  |  |
| **24** | MR PEAK VELOCITY | MR peak velocity |  |  |  |  |  |
| **25** | PV MEAN VELOCITY | across the pulmonary valve mean recorded velocity | mean velocity across the pulmonary valve | pulmonary valve mean velocity | pulmonary valve mean recorded velocity | PV mean velocity | PV mean recorded velocity |
| **26** | MR MEAN VELOCITY | MR mean Velocity |  |  |  |  |  |
| **27** | AV MEAN VELOCITY | across the aortic valve mean recorded velocity | mean velocity across the bioprosthetic aortic valve | mean recorded velocity across the bioprosthetic aortic valve | across the bioprosthetic aortic valve mean recorded velocity | Aortic valve mean recorded velocity | Aortic Valve mean Velocity |
| **27** | AV MEAN VELOCITY | AV mean recorded velocity | mean transaortic valve velocity | mean aortic valve velocity | AV mean velocity |  |  |
| **28** | MV MEAN VELOCITY | across the mitral valve mean recorded velocity | mean velocity across the bioprosthetic mitral valve | mean recorded velocity across the bioprosthetic mitral valve | across the bioprosthetic mitral valve mean recorded velocity | Mitral valve mean recorded velocity | Mitral Valve mean Velocity |
| **28** | MV MEAN VELOCITY | MV mean recorded velocity | mean transmitral valve velocity | mean mitral valve velocity | MV mean velocity |  |  |
| **29** | TV MEAN VELOCITY | TV mean velocity | Tricuspid Valve mean velocity |  |  |  |  |
| **30** | AORTIC STENOSIS | Aortic Stenosis | Aortic Valve Stenosis |  |  |  |  |
| **31** | MITRAL STENOSIS | Mitral Stenosis | Mitral Valve Stenosis |  |  |  |  |
| **32** | TRICUSPID STENOSIS | Tricuspid Stenosis | Tricuspid Valve Stenosis |  |  |  |  |

| **S.No** | **Data Elements** | **Synonymous Terminologies** | | | | | |
| --- | --- | --- | --- | --- | --- | --- | --- |
| **33** | DIMENSIONAL INDEX | Dimensionless Index |  |  |  |  |  |
| **34** | MVA(P1/2T) | MVA(P1/2T) (in cm) |  |  |  |  |  |
| **35** | PULMONARY ARTERY PRESSURE | Pulmonary Artery Pressure | Pulmonary Artery Systolic Pressure |  |  |  |  |
| **36** | RIGHT ARTERY PRESSURE | Right Atrial Pressure | RA pressure |  |  |  |  |
| **37** | MITRAL REGURGITATION | MR | MI | Mitral Regurgitation | Mitral Insufficiency |  |  |
| **38** | VENA CONTRACTA | Vena Contracta |  |  |  |  |  |
| **39** | REGURGITANT FRACTION | Regurgitant Fraction |  |  |  |  |  |
| **40** | REGURGITANT ORIFICE AREA | Effective regurgitant orifice area | effective orifice area | effective regurgitant orifice | regurgitant orifice area |  |  |
| **41** | AR PRESSURE HALF TIME | AR pressure half time | AI pressure half time | aortic regurgitation pressure half time | aortic insufficiency pressure half time |  |  |
| **42** | MV PRESSURE HALF TIME | MV pressure half time (in MS) |  |  |  |  |  |
| **43** | AORTIC FLOW REVERSAL | holodiastolic flow reversal in the descending aorta | holodiastolic aortic flow reversal in the descending aorta | diastolic flow reversal in the descending aorta | descending aorta reveals reversal of flow during much of diastole | aortic regurgitation, with holodiastolic flow reversal |  |
| **44** | LVEF | LVEF | EF | LV ejection fraction | left ventricular ejection fraction | left ventricle ejection fraction | ejection fraction |
| **44** | LVEF | EF(sp4-el) | EF(mod-sp2) |  |  |  |  |
| **45** | RVEF | RVEF | RV ejection fraction | Right ventricular ejection fraction | Right ventricle ejection fraction |  |  |

| **S.No** | **Data Elements** | **Synonymous Terminologies** | | | | | |
| --- | --- | --- | --- | --- | --- | --- | --- |
| **46** | DIASTOLIC FUNCTION | Diastolic Dysfunction | Diastolic function |  |  |  |  |
| **47** | E/A RATIO | E/A RATIO | E to A ratio |  |  |  |  |
| **48** | LV FILLING PRESSURE | LV filling pressure | Left Ventricle filling pressure/pressures | Left Ventricular filling pressure/pressures |  |  |  |
| **49** | E/e' Ratio | E/e' ratio | E to e' ratio | E:e' ratio |  |  |  |
| **50** | LVEDD | LVEDD | LV size end diastole | LV size-end diastole |  |  |  |
| **51** | LVESD | LVSD | LVESD | LV size end systole | LV size-end systole |  |  |
| **52** | BASAL TYPE | Basal septal hypertrophy |  |  |  |  |  |
| **53** | DEGREE OF LV HYPERTROPHY | Left ventricle hypertrophy | lv hypertrophy | LVH |  |  |  |
| **54** | DEGREE OF BS HYPERTROPHY | basal hypertrophy |  |  |  |  |  |
| **55** | SEPTAL THICKNESS | septal thickness |  |  |  |  |  |
| **56** | ATRIAL ENLARGEMENT | Atrial Enlargement | LA enlargement | Left atrial enlargement |  |  |  |
| **57** | LA DIMENSION | LA dimension | LA diameter |  |  |  |  |
| **58** | VOLUMN INDEX | LA Volume Index |  |  |  |  |  |
| **59** | BODY SURFACE AREA | BSA |  |  |  |  |  |
| **60** | RVOT VTI | RVOT Stroke Volume | RVOT VTI | RVOT velocity time integral |  |  |  |
| **61** | LVOT DIA | LVOT Dia | LVOT Diameter | LVOT dimension |  |  |  |
| **62** | AO ROOT DIA | AO Root Dia | Aortic Root Dia | Aortic Root Diameter | Aortic Root measured | Aortic Root dimension |  |
| **63** | AV VTI | AV Stroke Volume | AV VTI | AV velocity time integral | AO V2 VTI |  |  |
| **64** | MV VTI | MV Stroke Volume | MV VTI | MV velocity time integral | MV V2 VTI |  |  |

| **S.No** | **Data** **Elements** | **Synonymous Terminologies** | | | | | |
| --- | --- | --- | --- | --- | --- | --- | --- |
| **65** | LVOT VTI | LVOT stroke Volume | LVOT VTI | LVOT velocity time integral | V1 VTI |  |  |
| **66** | RVOT DIMENSION | RVOT diameter | ROT dimension |  |  |  |  |
| **67** | RV basal thickness | RV basal | Basal RV |  |  |  |  |
| **68** | TAPSE | TAPSE |  |  |  |  |  |
| **69** | RA SIZE | RA | Right Atrium |  |  |  |  |
| **70** | DEGREE OF RV HYPERTROPHY | Right ventricle hypertrophy | RV hypertrophy | RVH |  |  |  |
| **71** | TRICUSPID REGURGITATION | Tricuspid Insufficiency | Tricuspid Regurgitation |  |  |  |  |
| **72** | LA VOLUME | LA VOL | LA VOLUME |  |  |  |  |
| **73** | PERICARDIUM SIZE | Pericardium size | Pericardial Size |  |  |  |  |
| **74** | INFERIOR VENA CAVA | Inferior Vena Cava | IVC |  |  |  |  |
| **75** | RIGHT ATRIAL PRESSURE | Right Atrial Pressure | RA pressure |  |  |  |  |
| **76** | PULMONARY REGUGITATION | Pulmonary Insufficiency | Pulmonary Regurgitation |  |  |  |  |
| **77** | RVOT PEAK GRADIENT | RVOT peak pressure gradient | RVOT peak gradient | RVOT with….peak pressure gradient | LVOT with…peak gradient | LVOT peak pressure gradient |  |
| **78** | PULMONARY STENOSIS | Pulmonary Stenosis | Pulmonary Valve Stenosis |  |  |  |  |
| **79** | LVOT MEAN VELOCITY | across the LVOT mean recorded velocity | mean recorded velocity across the LVOT | LVOT mean velocity | LVOT mean recorded velocity | LVOT mean recorded velocity |  |
| **80** | AORTIC REGURGITAION | AR | AI | Aortic Regurgitation | Aortic Insufficiency |  |  |
