# Supplementary material for: A Natural Language Processing Tool for Large-Scale Data Extraction from Echocardiography Reports
Source: PLoS One. 2016 Apr 28;11(4):e0153749. doi: 10.1371/journal.pone.0153749 (PMC4849652; doi:10.1371/journal.pone.0153749)
Supplement: S2 Table — This table lists the accuracy (precision, recall, specificity, negative predictive value and F-1 score) of the eighty data elements extracted by EchoInfer. (DOC) [file pone.0153749.s002.doc]

S2 Table: Accuracy of eighty data elements extracted during this study.

| **Concept** | **Precision %** | **Recall %** | **Specificity %** | **NPV %** | **F-1 Score %** |
| --- | --- | --- | --- | --- | --- |
| Overall | 94.06 | 92.21 | 96.53 | 96.01 | 93.12 |
| LVEF | 95.65 | 93.62 | 50.00 | 40.00 | 94.62 |
| AO ROOT DIAMETER | 97.67 | 95.45 | 85.71 | 0.75 | 96.55 |
| AV MEAN GRADIENT | 95.12 | 92.86 | 77.78 | 70.00 | 93.98 |
| MITRAL REGURGITATION | 93.02 | 95.24 | 66.67 | 75.00 | 94.12 |
| MITRAL LEAFLET | 97.37 | 94.87 | 91.67 | 84.62 | 96.10 |
| BODY SURFACE AREA | 97.37 | 97.37 | 92.31 | 92.31 | 97.37 |
| AORTIC REGURGITATION | 94.12 | 91.43 | 87.50 | 82.35 | 92.75 |
| AV PEAK GRADIENT | 93.75 | 96.77 | 90.00 | 94.74 | 95.24 |
| AV PEAK VELOCITY | 93.33 | 90.32 | 90.00 | 85.71 | 91.80 |
| TR PEAK VELOCITY | 96.43 | 93.10 | 95.45 | 91.30 | 94.74 |
| TR PEAK GRADIENT | 96.30 | 92.86 | 95.65 | 91.67 | 94.55 |
| LVOT DIAMETER | 96.29 | 96.29 | 95.83 | 95.83 | 96.29 |
| AV VTI | 96.00 | 96.00 | 96.15 | 96.15 | 96.00 |
| MITRAL STENOSIS | 96.00 | 92.31 | 96.00 | 92.31 | 94.12 |
| AORTIC STENOSIS | 91.67 | 88.00 | 92.31 | 88.89 | 89.80 |
| CONCENTRIC TYPE | 95.83 | 92.00 | 96.15 | 92.59 | 93.88 |
| DEGREE OF LV HYPERTROPHY | 95.65 | 91.67 | 96.30 | 92.86 | 93.62 |
| AORTIC LEAFLET | 95.65 | 95.65 | 96.43 | 96.43 | 95.65 |
| AORTIC VALVE AREA | 95.24 | 90.91 | 96.55 | 93.33 | 93.02 |
| MV MEAN GRADIENT | 94.74 | 94.74 | 96.88 | 96.88 | 94.74 |
| MV VTI | 93.33 | 96.55 | 97.26 | 98.61 | 94.91 |
| LA DIMENSION | 94.12 | 88.89 | 96.97 | 94.12 | 91.43 |

S2 continued.

| **Concept** | **Precision %** | **Recall %** | **Specificity %** | **NPV %** | **F-1 Score %** |
| --- | --- | --- | --- | --- | --- |
| AV MEAN VELOCITY | 88.89 | 94.12 | 94.12 | 96.97 | 91.43 |
| MR PEAK VELOCITY | 92.86 | 92.86 | 97.30 | 97.30 | 92.86 |
| E/A RATIO | 100.00 | 100.00 | 100.00 | 100.00 | 100.00 |
| PASP | 100.00 | 100.00 | 100.00 | 100.00 | 100.00 |
| LVOT PEAK GRADIENT | 100.00 | 100.00 | 100.00 | 100.00 | 100.00 |
| LVOT PEAK VELOCITY | 100.00 | 100.00 | 100.00 | 100.00 | 100.00 |
| MVA(P1/2T) | 100.00 | 100.00 | 100.00 | 100.00 | 100.00 |
| LVOT STROKE VOLUME | 100.00 | 90.90 | 100.00 | 97.56 | 95.23 |
| MITRAL VALVE AREA | 90.91 | 90.91 | 97.50 | 97.50 | 90.91 |
| RIGHT ARTERY PRESSURE | 100.00 | 100.00 | 100.00 | 100.00 | 100.00 |
| VOLUMN INDEX | 90.91 | 90.91 | 97.50 | 97.50 | 90.91 |
| LVOT SROKE VOL | 100.00 | 100.00 | 100.00 | 100.00 | 100.00 |
| MV PEAK GRADIENT | 88.89 | 88.89 | 97.62 | 97.62 | 88.89 |
| LV FILLING PRESSURE | 100.00 | 100.00 | 100.00 | 100.00 | 100.00 |
| RVOT PEAK VELOCITY | 100.00 | 100.00 | 100.00 | 100.00 | 100.00 |
| PV PEAK GRADIENT | 100.00 | 100.00 | 100.00 | 100.00 | 100.00 |
| PV PEAK VELOCITY | 100.00 | 100.00 | 100.00 | 100.00 | 100.00 |
| RVOT PEAK GRADIENT | 80.00 | 66.67 | 97.78 | 95.65 | 72.73 |
| PRESSURE HALF | 100.00 | 100.00 | 100.00 | 100.00 | 100.00 |
| MV PEAK VELOCITY | 100.00 | 100.00 | 100.00 | 100.00 | 100.00 |
| REGURGITANT ORIFICE AREA | 100.00 | 100.00 | 100.00 | 100.00 | 100.00 |
| BASAL TYPE | 100.00 | 100.00 | 100.00 | 100.00 | 100.00 |

S2 continued.

| Concept | Precision % | Recall % | Specificity % | NPV % | F-1 Score % |
| --- | --- | --- | --- | --- | --- |
| DEGREE OF BASAL HYPERTROPHY | 100.00 | 100.00 | 100.00 | 100.00 | 100.00 |
| TRICUPSID MEAN GRADIENT | 100.00 | 100.00 | 100.00 | 100.00 | 100.00 |
| DIMENSIONAL INDEX | 100.00 | 100.00 | 100.00 | 100.00 | 100.00 |
| MR PEAK GRADIENT | 100.00 | 100.00 | 100.00 | 100.00 | 100.00 |
| TV PEAK GRADIENT | 100.00 | 100.00 | 100.00 | 100.00 | 100.00 |
| VENA CONTRACTA | 100.00 | 100.00 | 100.00 | 100.00 | 100.00 |
| AORTIC FLOW REVERSAL | 100.00 | 50.00 | 100.00 | 98.00 | 66.67 |
| DIASTOLIC FUNCTION | 100.00 | 100.00 | 100.00 | 100.00 | 100.00 |
| E/E' RATIO | 100.00 | 100.00 | 100.00 | 100.00 | 100.00 |
| RVOT SROKE VOL | 100.00 | 100.00 | 100.00 | 100.00 | 100.00 |
| LVEDD | 100.00 | 100.00 | 100.00 | 100.00 | 100.00 |
| LVESD | 100.00 | 100.00 | 100.00 | 100.00 | 100.00 |
| SEPTAL THICKNESS | 100.00 | 100.00 | 100.00 | 100.00 | 100.00 |
| TAPSE | 100.00 | 100.00 | 100.00 | 100.00 | 100.00 |
| RVEF | 88.23 | 93.75 | 94.28 | 97.05 | 90.90 |
| LA VOLUME | 87.5 | 93.33 | 97.70 | 98.83 | 90.32 |
| MV HALF PRESSURE TIME | 80.00 | 88.89 | 97.8 | 98.9 | 84.2 |
| RA PRESSURE (normal, large, small) | 87.5 | 93.33 | 97.70 | 98.8 | 90.3 |
| RIGHT ATRIUM | 97.6 | 98.8 | 88.2 | 93.75 | 98.24 |
| RV HYPERTROPHY | 100.00 | 100.00 | 100.00 | 100.00 | 100.00 |
| PULMONARY REGURGITATION | 100.00 | 100.00 | 100.00 | 100.00 | 100.00 |
| TV PEAK VELOCITY | 100.00 | 100.00 | 100.00 | 100.00 | 100.00 |

S2 continued.

| Concept | Precision % | Recall % | Specificity % | NPV % | F-1 Score % |
| --- | --- | --- | --- | --- | --- |
| TRICUSPID REGURGITATION | 92.30 | 94.73 | 72.72 | 80.0 | 93.50 |
| TRICUSPID STENOSIS | 91.66 | 95.65 | 97.44 | 98.71 | 93.61 |
| ATRIAL ENLARGEMENT | * | * | * | * | * |
| PV MEAN GRADIENT | * | * | * | * | * |
| PV MEAN VELOCITY | * | * | * | * | * |
| MR MEAN VELOCITY | * | * | * | * | * |
| MV MEAN VELOCITY | * | * | * | * | * |
| TV MEAN VELOCITY | * | * | * | * | * |
| REGURGITANT FRACTION | * | * | * | * | * |
| RVOT DIMENSION | * | * | * | * | * |
| BASAL RV SIZE | * | * | * | * | * |
| PERICARDIAL SIZE | * | * | * | * | * |
| INFERIOR VENA CAVA | * | * | * | * | * |
| PULMONIC STENOSIS | * | * | * | * | * |
| TV MEAN VELOCITY | * | * | * | * | * |
| REGURGITANT FRACTION | * | * | * | * | * |

*Asterisk mark indicates that the data element is not found in the test set of 50 echocardiography reports.
