# Supplementary material for: A Natural Language Processing Tool for Large-Scale Data Extraction from Echocardiography Reports
Source: PLoS One. 2016 Apr 28;11(4):e0153749. doi: 10.1371/journal.pone.0153749 (PMC4849652; doi:10.1371/journal.pone.0153749)

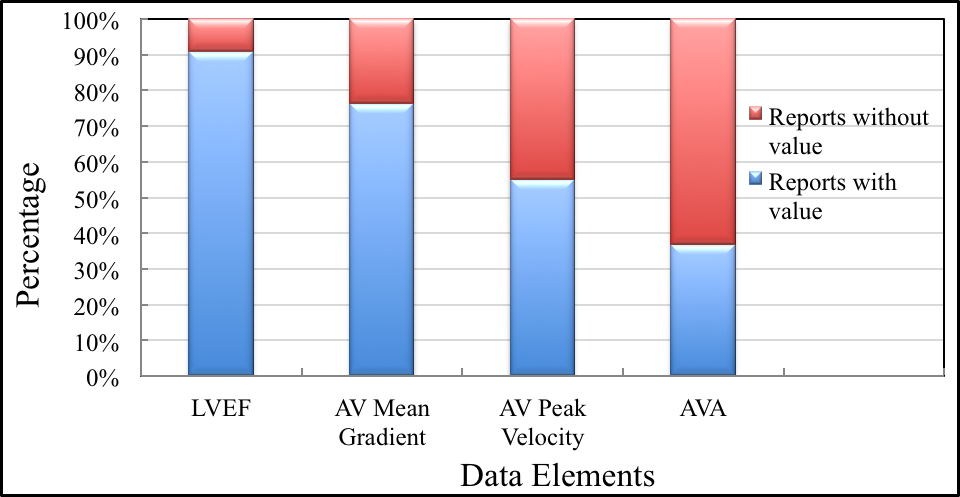
S3 Table: Breakdown of percentage of echocardiographic reports lacking any mention of various data elements as determined by EchoInfer.


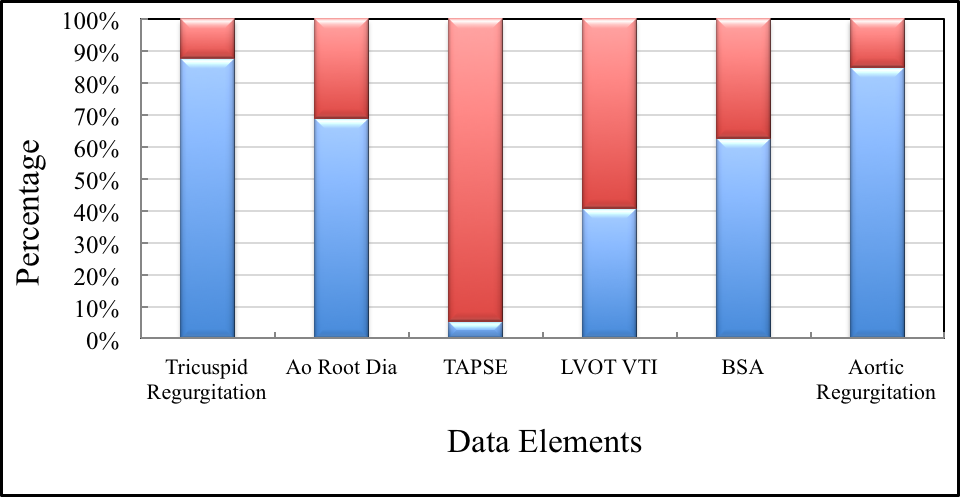

Supplement: S3 Table — This figure shows the relative proportion of Echo reports that miss ten of eighty data elements. (DOC) [file pone.0153749.s003.doc]
